# Mapping competency in public health training – experience of the Europubhealth consortium

**DOI:** 10.1186/s12909-023-05010-9

**Published:** 2024-01-10

**Authors:** Olivier Grimaud, Mathilde Foucrier, Kasia Czabanowska, for the Europubhealth Consortium, Sarah Barnes, Ariane Bauernfeind, Timo Clemens, Mary Codd, Anne-Françoise Donneau, Christoph Sowada, Catherine Keller, Aurore Gely-Pernot, Judith Mueller, Marie-Renée Guevel, Florence Bodeau-Livinec, Laurence Théault, Olivier Grimaud, Kasia Czabanowska

**Affiliations:** 1 Arènes-UMR 6051, RSMS-U 1309, EHESP, CNRS, Inserm, Rennes, France; 2 International Relations department, EHESP, Rennes, France; 3 INTHEALTH, CAPHRI, FHML, Maastricht University, Maastricht, The Netherlands

**Keywords:** Education, Public health, Competency

## Abstract

**Background:**

Public health education aims at producing a competent workforce. The WHO-ASPHER framework proposes a set of relevant public health competencies organised in 10 sections (e.g. science practice, leadership, law policies and ethics etc). As part of the Europubhealth (EPH) consortium, eight universities collaborate for the delivery of a 2-year international public health master course. The training pathway includes a first “foundation” year, with a choice of four options (components), and a second “specialisation” year with a choice of seven components. In 2020, EPH consortium decided to use the WHO-ASPHER framework in order to map the competencies addressed and the level of proficiency targeted by each component of its master course.

**Methods:**

An 84-item questionnaire covering the whole WHO-ASPHER framework was sent to the 11 EPH component coordinators, asking them to rate the proficiency levels targeted at the end of their courses. Answers from each coordinator were summarised by calculating mean proficiency levels for each of the 10 competency sections. We used Bland & Altman plots to explore heterogeneity of answers and then calculated transformed scores to account for rating heterogeneity. We use tabulation and a heat map to explore patterns of proficiency levels across components.

**Results:**

There were differences in overall proficiency levels between years with, as expected, higher scores in year two. Year one components reached medium to high proficiency scores for the sections “science practice”, “health promotion” and “communication” with scores ranging from 2.6 to 3 (on a 1-low to 4-high scale). When compared with year one on a heat-map, year two components displayed more contrasted profiles, typically aiming for high proficiency level (i.e. scores above 3.5) on 3 out of the 10 sections of competencies. Except for the “collaborations and partnership” section, the training pathways offered by the EPH master course seem to offer opportunities for a high proficiency level in all domains of competencies.

**Conclusions:**

The mapping proved a useful exercise to identify strengths and complementarities among the EPH consortium. The results suggest that the EPH master course is coherent and offers students opportunities to gain proficiency in most competencies relevant to public health practice.

**Supplementary Information:**

The online version contains supplementary material available at 10.1186/s12909-023-05010-9.

## Background

The main purpose of public health education is to equip future professionals with the necessary competencies to maintain and improve population health. In the last decades, works have been ongoing to define the set of competencies requested for public health practice. Competencies are composites of individual attributes (i.e., knowledge, skills, and attitudinal or personal aspects, etc.) that represent context-bound productivity [1, 2] necessary for the practice of public health. As such, competencies transcend the boundaries of specific disciplines and provide the building blocks for effective public health practice and the application of an overall public health approach. Public health employers may use the competency approach for identifying gaps in the workforce, designing job descriptions and more generally for supporting resource management. The competency approach may also guide the development of education and training programmes [3].

Since 2006 eight European universities collaborate in order to deliver the Europubhealth (EPH) Master. The EPH consortium and Master program have been described extensively elsewhere [4, 5]. In brief, the 2 years Master course consists in: a “first year component”, or “foundation year”, whose aim is the acquisition of core public health knowledge and competencies; a “second year component” or “specialisation year”, when more proficiency in a specific field, such as health promotion or environmental health, is targeted (see Table 1 for a description of all components). The course is supported by the Erasmus Mundus program of the European Commission, thus attracting students from all over the world.

**Table 1:** Components of the Europubhealth Public Health Master

	Component title	University	Town, country
**Year 1**	Core competencies in public health	University College of Dublin	Dublin, Ireland
	“	Andalusian School of public health – University of Granada	Granada, Spain
	“	University of Liège	Liège, Belgium
	“	School of Health and related research	Sheffield, UK
**Year 2**	Management of health services	Andalusian School of public health – University of Granada	Granada, Spain
	Governance of health systems in transition	Institute of Public Health – Jagiellonian University	Krakow, Poland
	Leadership in European Public Health	Maastricht University	Maastricht, Netherlands
	Environmental and occupational health sciences	French School of Public Health	Paris, France
	Advanced biostatistics and epidemiology	French School of Public Health	Paris, France
	Law and Public health	University of Rennes 1	Rennes, France
	Health promotion and prevention	French School of Public Health	Rennes, France

Right from the inception of the EPH consortium, members have examined commonalities and specificities of each component curricula. The aim being to ensure that every possible pathway led to the acquisition of sufficient level of knowledge and competencies. The consortium decided to use the 2020 WHO-ASPHER framework [6] to map the competencies addressed in the different components proposed in the EPH Master and to estimate the proficiency level targeted.

## Method

A survey using an adapted version of the WHO-ASPHER framework was carried out among EPH consortium members. This framework was developed through literature review and exchanges between a wide array of stakeholders [6, 7]. It is organised in 10 broad domains of competencies, each of them delineated by a set of 6 to 12 detailed items (see Table 2, and supplementary material for the full list of items). The first adaptation consisted in adding the sentence “To which extent does the course enable students to develop this competency?” to each of the 84 items of the framework. Consortium members were asked to assess the proficiency level that students are expected to reach at the end of their course. The proficiency scale in the original framework contained five levels (see Table 3). The second adaptation consisted in excluding the fifth option “expert” as a possible answer, on the ground that this level can only be attained through professional experience. The academic coordinator of each component was responsible for gathering the responses related to its own teaching program. This typically meant consulting the faculty involved in the respective teaching modules. The survey was launched in December 2020. Answers from each of the 11 components were returned electronically by July 2021.

**Table 2:** The WHO-ASPHER competency framework

Competency domain	Nb of items	Example of item
Science practice	10	Knows how to retrieve, analyse and appraise evidence from all data sources to support decision making
Promoting Health	8	Fosters citizen empowerment and engagement within the community
Law Policies and Ethics	6	Knows, understands and applies the relevant international, European and national laws or regulations to maximise opportunities to protect and promote health and wellbeing
One Health and Health security	12	Understands the local implications of the One Health approach, its global interconnectivity and its impact on health conditions in the population
Leadership and system thinking	9	Effectively leads interdisciplinary teams to work in a coordinated manner in different areas of public health practice
Collaborations and partnerships	6	Identifies, connects and manages relationships with stakeholders in interdisciplinary and inter-sectorial projects to improve public health services and achieve public health goals
Communication culture and advocacy	8	Communicates strategically by defining the target audience, listening and developing audience-appropriate messaging
Governance and resource management	10	Effectively applies knowledge of organisational systems, theories and behaviours in order to prioritise, align and deploy all relevant resources towards clear strategic goals and objectives
Professional Development and Ethical reflexive Pratice	7	Acts according to ethical standards and norms with integrity, promotes professional accountability, social responsibility and the public good
Organisational literacy and adaptability	8	Actively prepares and adapts to changing professional environments and circumstances

**Table 3:** Proficiency levels of the WHO-ASPHER competency framework.

1. **Novice** Novices have little or no knowledge/ability or no previous experience of the competency described and need close supervision or instruction. 2. **Advanced beginner** Advanced beginners have some knowledge of the competency described, but there are gaps in their knowledge, and they would not be able to apply that knowledge in a sustained way to complete a work task. 3. **Competent** Competent persons can troubleshoot problems on their own and when supported by experts may begin to figure out how to solve novel problems. Competent persons would have an adequate level of competence to undertake work tasks in this area, albeit under the supervision of a more experienced professional. 4. **Proficient** Proficient persons deal with complex situations holistically. They will be able to take full responsibility for own work and coach others. Proficient persons have detailed knowledge and would feel confident to undertake work tasks in this area, without supervision. 5. **Expert** Experts are the primary sources of knowledge and information in any field. They holistically grasp complex situations and move between intuitive and analytical approaches with ease. Experts will have a great deal of expertise in the particular competency and others may come to them for advice.	

In order to summarise the answers, we collapsed the scores into average scores for each competency domains. This created a table of 10 competency average scores for each of the 11 EPH components. Bland & Altman plots [8] suggested heterogeneity of answers particularly within year one, with one component rating systematically lower level of proficiencies. This was in clear contrast with the consortium experience of a fairly equivalent overall level of proficiencies whatever year one pathway. In order to address this inter-rater variability, scores were transformed so that the mean across competencies proficiency level were similar for all 4 year one components (see Supplementary material for detailed method). Average scores for the 11 year two components were transformed similarly.

Several options were considered for displaying the results in tabular or graphical formats. In this paper, we choose the heat map in order to highlight similarities and contrasts of competency levels across components. In order to compare variations across competencies, components and year, we used quartiles of the overall distribution of transformed scores as limit for the four shadings of the heat map (from lighter = lower level, to darker = higher level of competency). We also use radar graphs in order to illustrate levels of proficiency aimed at for several combinations of year one and Year two components.

## Result

Table 4 shows the distribution of original and transformed competency scores for year one and year two components. Mean original scores (across the 10 competencies) vary from 1.9 to 2.7 in year one and from 2.0 to 3.0 in year two. Bland and Altman plots were suggestive of between raters variability with, for instance, year one original scores of Granada and Sheffield respectively systematically above and below year one components average (see Supplementary material). Table 4 also shows how transformed scores erase these differences across partners, while maintaining a range of proficiency levels across competencies comparable to that of original scores’range.

**Table 4:** Distributions of original and transformed scores across Year 1 and Year 2 components of the Europubhealth Public Health Master

	Original score			Transformed score		
	min	mean	max	min	mean	max
Year 1						
Dublin	2.2	2.6	3.0	1.9	2.4	2.8
Granada	2.3	2.7	3.0	2.0	2.4	2.7
Liège	1.6	2.3	3.0	1.7	2.4	3.1
Sheffield	1.3	1.9	2.5	1.8	2.4	3.0
Year 2						
Granada management	2.6	3.0	4.0	2.1	2.5	3.5
Krakov, governance	1.3	2.4	3.9	1.3	2.5	4.0
Maastricht, leardership	1.8	3.0	4.0	1.3	2.5	3.6
Paris, environment	1.3	2.1	3.8	1.7	2.5	4.0
Paris, epidemiology	1.3	2.0	4.0	1.8	2.5	4.0
Rennes, law	1.8	2.5	4.0	1.9	2.5	4.0
Rennes, health promotion	2.0	2.6	4.0	1.9	2.5	3.9

As displayed by the heat map (Table 5), results from year one are fairly homogeneous inasmuch as the domains “Science practice”, “Promoting health”, and “Communication …” reached medium to high scores in all components, whereas “One health…”, “Governance…” and “Collaborations…” scored uniformly low. The former group of high scoring domains are closely related to the scientific foundations of public health (e.g. epidemiology, health promotion).

**Table 5:**
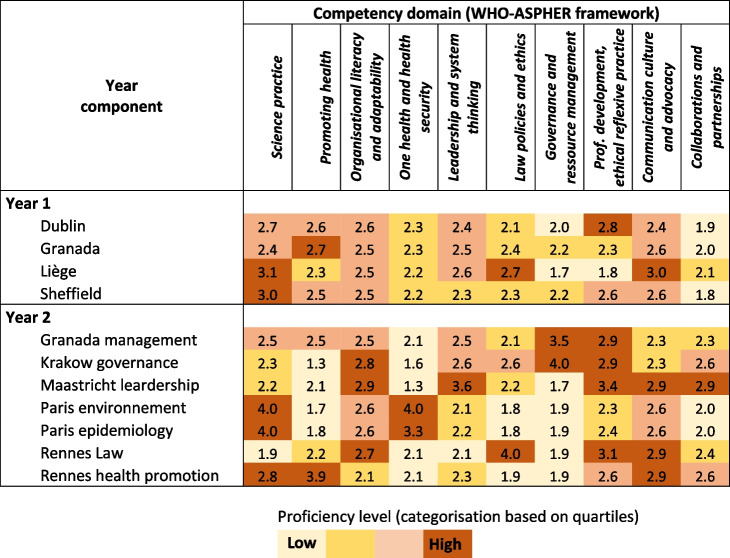
Heat map presenting proficiency levels aimed at for public health competencies at the end of Year 1 and Year 2 components of the Europubhealth Master

Year two mean proficiency levels are slightly higher than that of year one (2.5 versus 2.4, Table 4). However, compared with year one, results for year two on the heat map show a more contrasted pattern with domains reaching very high scores when they defines the component’s specialty. Examples include the competency domain “law” with high score from Rennes law, “leardership” for the Maastricht and “Governance” for Krakow. In contrast, “organisational literacy…”, and to a lesser extent “professional development …” and “communication …”, are attributed medium to high scores across year two components. When scanning horizontally year two scores, most components focus on two or three domains of competencies, thus in accordance with the specialisation vocation of the second stage of the training. At first glance, this does not apply to Maastricht and Rennes Law for whom at least four competency domains reach the highest level. However close inspection of the scores still suggests a strong polarisation on their defining domain, i.e. respectively Leadership and Law.

**Fig. 1:**
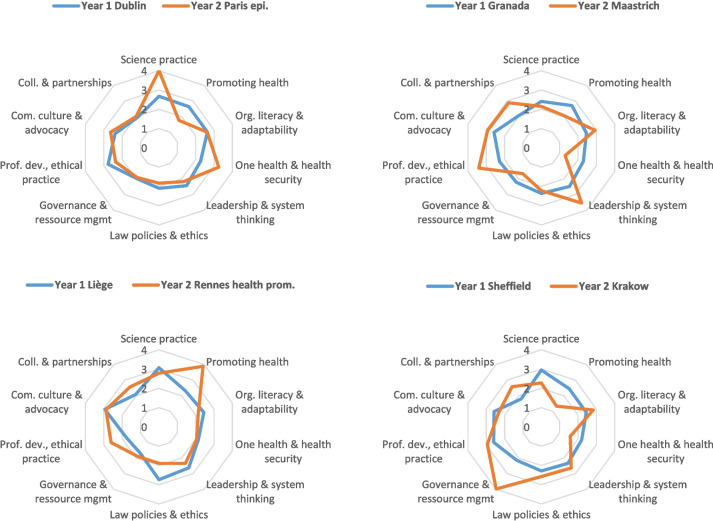
Graphical illustration of proficiency levels aimed at for four combinations of Year 1 and Year 2 components of the Europubhealth Master

The radar graph (Fig. 1) shows the “shapes” of curricula for four combinations of year one and year two components. This illustrates the “all-round pattern of medium proficiency level” proposed by the 4 year one components. In comparison, the sample of year two components displays angular shapes, pointing towards a specific domain of specialisation.

## Discussion

Using the WHO-ASPHER competency framework, this survey undertaken by the EPH consortium showed that members proposing year one components (first year of master training) endeavour to equip students with a medium-high level of proficiency across a range of “foundational” competencies. These include scientific disciplines such as epidemiology, communication, and the application domain of health promotion. Taken together, these contents should allow students to understand and embrace the population perspective, and the related concepts of health determinants, which underpins public health practice and research [9]. In contrast, year two components aim at a high proficiency level on one or few competencies. While some year two pathways still aim at the “foundational” competencies mentioned before, others focus on additional sciences and domains, such as management, governance or leadership. It should be noted that, based on the responses from the academic coordinators, the range of specialisations offered by the EPH consortium covers all 10 areas of the WHO-ASPHER competency framework.

We identify several benefits for carrying out the competency mapping. First, the survey helped to delineate accurate profiles of each EPH Master’s component. Thus, scores for the 4 Year one components suggest medium level of proficiency in competencies associated with public health foundation disciplines. They nevertheless highlight some meaningful variations across members. For instance, the higher score assigned to the competency “Promoting health” by Granada compared with other Year one components is in line with the focus of the training offered by this member. Another benefit of the mapping exercise is that the heat map and spider graphs could prove useful communication “by-products” to assist future candidates in choosing the specific pathway which most closely match their aspiration. Finally, we argue that engaging in the mapping exercise as a consortium of international universities has proved a useful means of enhancing knowledge and trust among all members.

Competency frameworks are multipurpose tools that can be used to assess current and to plan for future workforce. They can also prove useful in designing and assessing educational curricula^3^. A number of public health competency frameworks have been developed, some of them within specific national or regional contexts [10–12], others with a specific focus (e.g. on leadership [13], or communicable diseases surveillance and control [14]). Being a general, recently developed and Europe grounded framework were features that guided our choice towards the WHO-ASPHER model. It is likely that the different versions of public health competency frameworks overlap in a considerable extent. However, public health practice is constantly evolving due to changes in health needs, scientific discoveries and technical innovations. We would therefore recommend to use a framework as up to date as possible for a purpose similar or related to ours in the survey.

A comprehensive and useful method for charting public health competencies to educational programs have been proposed by Neiworth et al. [15]. *“Resolving competency mapping inconsistencies”* is one of the six steps outlined in their method. Indeed, cultural differences as well as individual subjectivity are likely to influence responses. We confronted this issue since one of the Year one component reported systematically lower levels of proficiency compared with other Year one members. Discussion between members established that this was very much at odds with experience of the consortium, which pointed towards comparable levels of proficiency across Year one components. Following this exchange, we opted to transform arithmetically the scores to correct for what appeared to be an issue of calibration. An alternative option would have been to undertake a second or even several successive rounds (as in a Delphi consensus method). A further sophistication to the mapping would be to define and attribute “importance weight” to each of the 84 items of the framework, whereas we only calculated crude mean scores. Although such methodological alternatives are likely to improve the validity of the mapping, the expected benefits should be weighed against the extra time and resource required.

Applying a similar or adapted approach to evaluate the proficiency levels achieved by students at the end of their training appears as a logical next step. The EPH consortium is considering implementing these assessments in the near future, although option for implementation are still under consideration. The assessment procedures and examinations for each component could be cross-referenced with the relevant section of the WHO-ASPHER framework and, if necessary, adapted to provide an assessment of students’ progress in acquiring competences. Alternatively, or complementarily, a similar approach could be used in end of training assessment and employer surveys.

### Supplementary Information

**Additional file 1.**

## Data Availability

The database of mean original and transformed scores is available upon motivated request to the corresponding author.

## Abbreviations

EPH: Europubhealth Consortium
WHO-ASPHER: World Health Organisation – Association of Schools of Public Health in the European Region
