# Supplementary material for: Mapping competency in public health training – experience of the Europubhealth consortium
Source: BMC Med Educ. 2024 Jan 10;24:54. doi: 10.1186/s12909-023-05010-9 (PMC10782686; doi:10.1186/s12909-023-05010-9)
Supplement: Supplementary file 1 — Additional file 1. [file 12909_2023_5010_MOESM1_ESM.docx]

**Supplementary material**

- **Instrument used for the survey on public health competencies**
- **Figure S1** : **Bland and Altman plots (page 13)**
- **Method for transforming the scores (page 14)**

# Instrument used for the survey on public health competencies

Indicate your assessment of the proficiency level aimed at in your course for each item of the WHO-ASPHER public health competency framework using the following scale:

| Novice | 1 |
| --- | --- |
| Advanced Beginner | 2 |
| Competent | 3 |
| Proficient | 4 |

## Science practice

|  | Proficiency level |
| --- | --- |
| Knows the features of demographic structure in a given society/community and understands the process of demographic change and its implications for public health To which extent does the course enable students to develop this competency? |  |
| Is able to describe the key features of the epidemiology of the significant causes of morbidity and mortality in the population for which they have responsibility   To which extent does the course enable students to develop this competency? |  |
| Uses vital statistics and health indicators effectively to increase knowledge and generate evidence about population health, including within at-risk and vulnerable groups   To which extent does the course enable students to develop this competency? |  |
| Knows how to retrieve, analyse and appraise evidence from all data sources to support decision making   To which extent does the course enable students to develop this competency? |  |
| Has awareness of the health needs of the population based on considerations of burden of disease, indicators, characterisation of risks and demand/access for healthcare   To which extent does the course enable students to develop this competency? |  |
| Contributes to or leads community based health needs assessments ensuring that these assessments consider biological, social, economic, cultural, political physical determinants of health and wider determinants of health such as deprivation   To which extent does the course enable students to develop this competency? |  |
| Designs and conducts qualitative and/or quantitative research which builds on existing evidence and adds to the evidence base for public health practice, involving relevant stakeholders in this process   To which extent does the course enable students to develop this competency? |  |
| Evaluates local public health services and interventions applying sound methodology based on recognised evaluation models   To which extent does the course enable students to develop this competency? |  |
| Develops and implements standards, protocols and procedures which incorporate national and/or international “best practices” in the health system   To which extent does the course enable students to develop this competency? |  |
| Understands the health system structure, its governance, funding mechanisms and how healthcare services are organised   To which extent does the course enable students to develop this competency? |  |

## Promoting health

|  | Proficiency level |
| --- | --- |
| Knows and has intellectually incorporated the underlying concepts of the Ten Essential Public Health Operations (EPHOs) and understands their implications for own organisation, the partners, the public health system   To which extent does the course enable students to develop this competency? |  |
| Assesses the focus and scope of initiatives to promote health through assessment of need to achieve positive changes in individual and community health   To which extent does the course enable students to develop this competency? |  |
| Knows, supports and engages in and supports health promoting and health literacy activities and programmes for the implementation of good practice to promote health at a population level and specific organisation or institutional level   To which extent does the course enable students to develop this competency? |  |
| Uses evidence-based methods and strategies, social participation and inter-sectorial approaches as tools for promoting health and influencing public policies impacting health   To which extent does the course enable students to develop this competency? |  |
| Evaluates the effectiveness of activities to promote health geared toward producing changes at the community and individual level, in public or social policy to benefit health and quality of life   To which extent does the course enable students to develop this competency? |  |
| Fosters citizen empowerment and engagement within the community, developing capabilities that are valuable to actively participate in the development and decision making of a healthy community   To which extent does the course enable students to develop this competency? |  |
| Where needed, generates or promulgates factual information to counteract industry marketing in relation to: nutrition, tobacco cessation, alcohol consumption reduction, etc.   To which extent does the course enable students to develop this competency? |  |
| Knows the basis of secondary prevention and screening programmes   To which extent does the course enable students to develop this competency? |  |

## Law Policies and Ethics

|  | Proficiency level |
| --- | --- |
| Understands and addresses the upstream fundamental causes of health inequalities and downstream consequences (such as drug, alcohol abuse and smoking) in ensuring equitable access to health services   To which extent does the course enable students to develop this competency? |  |
| Knows, understands and applies the relevant international, European and national laws or regulations to maximise opportunities to protect and promote health and wellbeing.   To which extent does the course enable students to develop this competency? |  |
| Applies scientific principles and concepts to inform discussion of health-related, fiscal, administrative, legal, social and political issues in the workplace.   To which extent does the course enable students to develop this competency? |  |
| Compares and contrasts health and social service delivery systems among and between countries, which reflect diverse political, organizational and legal contexts, using these experiences to improve access, regulation and the fairness of health systems   To which extent does the course enable students to develop this competency? |  |
| Participates in the implementation of health and social policies and plans that help guarantee the right to equitable and effective healthcare and policies enabling environments favourable to health   To which extent does the course enable students to develop this competency? |  |
| Develops and implements strategies based on relevant evidence, legislation, emergency planning procedures, regulations and policies   To which extent does the course enable students to develop this competency? |  |

## One Health and Health security

|  | Proficiency level |
| --- | --- |
| Identifies and uses legislation, codes of ethical practice and standards that have an impact on public health professional practice in the interaction with individuals, organisations, and communities.   To which extent does the course enable students to develop this competency? |  |
| Understands the local implications of the One Health approach, its global interconnectivity and its impact on health conditions in the population   To which extent does the course enable students to develop this competency? |  |
| Critically analyses the changing nature, key factors and resources that shape One Health in order to influence actions (emergency preparedness planning and response) at the local and international level   To which extent does the course enable students to develop this competency? |  |
| Knows and where needed applies the International Health Regulations to coordinate and develop strategic partnerships and resources in key sectors and disciplines for health security purposes   To which extent does the course enable students to develop this competency? |  |
| Understands and promotes occupational safety and health, as a multidisciplinary field concerned with the safety, health, and welfare of people at work   To which extent does the course enable students to develop this competency? |  |
| Knows the practical principles of food safety essential to public health.   To which extent does the course enable students to develop this competency? |  |
| Understands the basics of vaccine preventable diseases and is able to advise on organisational aspects of vaccination activities   To which extent does the course enable students to develop this competency? |  |
| Knows and participates in the development and application of multi-sectorial  evidence-based guidelines and systems for surveillance, prevention and control of diseases and other acute public health events   To which extent does the course enable students to develop this competency? |  |
| Performs surveillance of risks and threats to the full continuum of factors that influence and determine health in order to identify intervention needs   To which extent does the course enable students to develop this competency? |  |
| Identifies minimum or basic safety conditions in health care delivery, for the design and implementation of programmes and activities for surveillance, risk management and sustainability inherent to health service delivery   To which extent does the course enable students to develop this competency? |  |
| Identifies and describes the environmental determinants of health and the connections between environmental protection and public health policy.   To which extent does the course enable students to develop this competency? |  |

## Leadership and system thinking

|  | Proficiency level |
| --- | --- |
| Knows and correctly identifies the main features of the climate change process, along with its implications for public health and understands the public health responsibility for the natural environment.   To which extent does the course enable students to develop this competency? |  |
| Inspires and motivates others to work towards a common vision, programme, and/or organizational goals   To which extent does the course enable students to develop this competency? |  |
| Acts as a role model, builds trust and demonstrates positive and engaging behaviour   To which extent does the course enable students to develop this competency? |  |
| Facilitates development of others as leaders   To which extent does the course enable students to develop this competency? |  |
| Clearly identifies and supports roles and responsibilities of all team members, including external stakeholders   To which extent does the course enable students to develop this competency? |  |
| Demonstrates emotional intelligence with an awareness of the impact of one’s own beliefs, values, and behaviours upon one’s own decision-making and upon the reactions of others   To which extent does the course enable students to develop this competency? |  |
| Demonstrates practicality, flexibility, and adaptability in the process of working with others, emphasizing the achievement of goals as opposed to rigid adherence to traditional and commonly used work methods   To which extent does the course enable students to develop this competency? |  |
| Effectively leads interdisciplinary teams to work in a coordinated manner in different areas of public health practice   To which extent does the course enable students to develop this competency? |  |
| Catalyses change (behavioural and/or cultural) in the organisation, communities and/or individuals   To which extent does the course enable students to develop this competency? |  |
| Understands the principles of systems thinking and is able to apply them within systematic inquiry to analyse, model and improve Public Health organisations and services at different strategic levels   To which extent does the course enable students to develop this competency? |  |

## Collaborations and partnerships

|  | Proficiency level |
| --- | --- |
| Works across sectors at the local/national/international level organisational structures   To which extent does the course enable students to develop this competency? |  |
| Understands the interdependency, integration, and competition among healthcare sectors and different actors who have interests in public health issues   To which extent does the course enable students to develop this competency? |  |
| Identifies, connects and manages relationships with stakeholders in interdisciplinary and inter-sectorial projects to improve public health services and achieve public health goals   To which extent does the course enable students to develop this competency? |  |
| Builds, maintains and effectively uses strategic alliances, coalitions, professional ntworks and partnerships to plan, generate evidence and implement programmes and services that share common goals and priorities to improve the health and wellbeing of populations   To which extent does the course enable students to develop this competency? |  |
| Evaluates partnerships and addresses barriers to successful collaboration in order to improve public health services   To which extent does the course enable students to develop this competency? |  |
| Understands and applies effective techniques for working with boards and governance structures including regulatory, professional and accreditation agencies   To which extent does the course enable students to develop this competency? |  |

## Communication culture and advocacy

|  | Proficiency level |
| --- | --- |
| Communicates and shares information and responsibility effectively at different organisational levels to gain political commitment, policy support, and social acceptance for a particular health goal or programme   To which extent does the course enable students to develop this competency? |  |
| Communicates strategically by defining the target audience, listening and developing audience-appropriate messaging   To which extent does the course enable students to develop this competency? |  |
| Communicates facts and evidence effectively within the context of translating science and evidence into practice and policy for various actors in the system and populations of concern in particular to increase the effectiveness of responses to risks, threats, and damages to health   To which extent does the course enable students to develop this competency? |  |
| Communicates health messages (including risks to health) in an effective way (both in writing and verbally) through a range of modern media and social marketing to lay, professional, academic and political audiences   To which extent does the course enable students to develop this competency? |  |
| Understands and applies cultural awareness and sensitivity in communication with diverse populations   To which extent does the course enable students to develop this competency? |  |
| Communicates with respect when representing professional opinions and encourages other team members, including community members and patients, to express their opinions and contribute to decision-making   To which extent does the course enable students to develop this competency? |  |
| Prepares and delivers outputs to facilitate communication within and between organisations such as meeting agendas, presentations, reports and project dissemination   To which extent does the course enable students to develop this competency? |  |
| Advocates for healthy public policies and services that promote and protect the health and well-being of individuals and communities   To which extent does the course enable students to develop this competency? |  |

## Governance and resource management

|  | Proficiency level |
| --- | --- |
| Understands and applies the principles of economic thinking in public health   To which extent does the course enable students to develop this competency? |  |
| Is proactive in designing and monitoring quality standards and applies quality improvement methods and tools to identify internal and external facilitators and barriers that may affect the delivery of the Ten Essential Public Health Operations   To which extent does the course enable students to develop this competency? |  |
| Effectively applies knowledge of organisational systems, theories and behaviours in order to prioritise, align and deploy all relevant resources towards clear strategic goals and objectives   To which extent does the course enable students to develop this competency? |  |
| Effectively manages people, most specifically by providing clarity on task responsibility, ensuring sufficient resources and training, and providing regular feedback on performance   To which extent does the course enable students to develop this competency? |  |
| Effectively plans the allocation of work tasks to achieve goals set by the organisation   To which extent does the course enable students to develop this competency? |  |
| Develops job descriptions to assure staffing at different organisational levels, conducts hiring interviews and evaluates candidates.   To which extent does the course enable students to develop this competency? |  |
| Demonstrates knowledge of basic business practices, such as terms of reference, business plans, contracting, and project management   To which extent does the course enable students to develop this competency? |  |
| Effectively uses key accounting principles and financial management tools, such as financial plans and measures of performance   To which extent does the course enable students to develop this competency? |  |
| Effectively uses risk management principles and programmes, such as risk assessment and analysis   To which extent does the course enable students to develop this competency? |  |
| Performs a health economic evaluation and assessment of a given procedure, intervention, strategy or policy   To which extent does the course enable students to develop this competency? |  |

## Professional Developement and Ethical reflexive Pratice

|  | Proficiency level |
| --- | --- |
| Demonstrates the willingness to pursue lifelong learning in the field of public health   To which extent does the course enable students to develop this competency? |  |
| Self-assesses and addresses own development needs based on career goals and required competencies   To which extent does the course enable students to develop this competency? |  |
| Acts according to ethical standards and norms with integrity, promotes professional accountability, social responsibility and the public good   To which extent does the course enable students to develop this competency? |  |
| Critically reviews, evaluates own practices in relation to public health principles including critical self-reflection   To which extent does the course enable students to develop this competency? |  |
| Acts upon and promotes evidence-based professional practice   To which extent does the course enable students to develop this competency? |  |
| Ensures availability of professional development opportunities   To which extent does the course enable students to develop this competency? |  |
| Demonstrates an ability to understand and manage conflict-of-interest situations as defined by organizational regulations, policies and procedures   To which extent does the course enable students to develop this competency? |  |

## Organisational literacy and adaptability

|  | Proficiency level |
| --- | --- |
| Shows entrepreneurial orientation through pro-activeness, innovativeness, risk-taking, generating potential solutions to critical situations and evaluating their feasibility   To which extent does the course enable students to develop this competency? |  |
| Demonstrates persistence, perseverance resilience and the ability to call upon personal resources and energy at times of threat or challenge   To which extent does the course enable students to develop this competency? |  |
| Is able to cope with uncertainty and to manage work-related stress   To which extent does the course enable students to develop this competency? |  |
| Actively prepares and adapts to changing professional environments and circumstances   To which extent does the course enable students to develop this competency? |  |
| Delivers tasks within limited time frame to be able to work with deadlines   To which extent does the course enable students to develop this competency? |  |
| Applies methodologies, (digital) technologies and good practices for the management, analysis and storage of data and health information   To which extent does the course enable students to develop this competency? |  |
| Understands and applies a range of relevant information technology tools, social media and software   To which extent does the course enable students to develop this competency? |  |
| Is aware of and knows how to apply for available funding sources and opportunities, responds to calls for projects, develops and submits project applications and grants, drafts tender and project briefs   To which extent does the course enable students to develop this competency? |  |

**Figure S1** : **Bland and Altman plots for Year 1 (top panel) and Year 2 (bottom panel) components of the Europubhealth Master.**


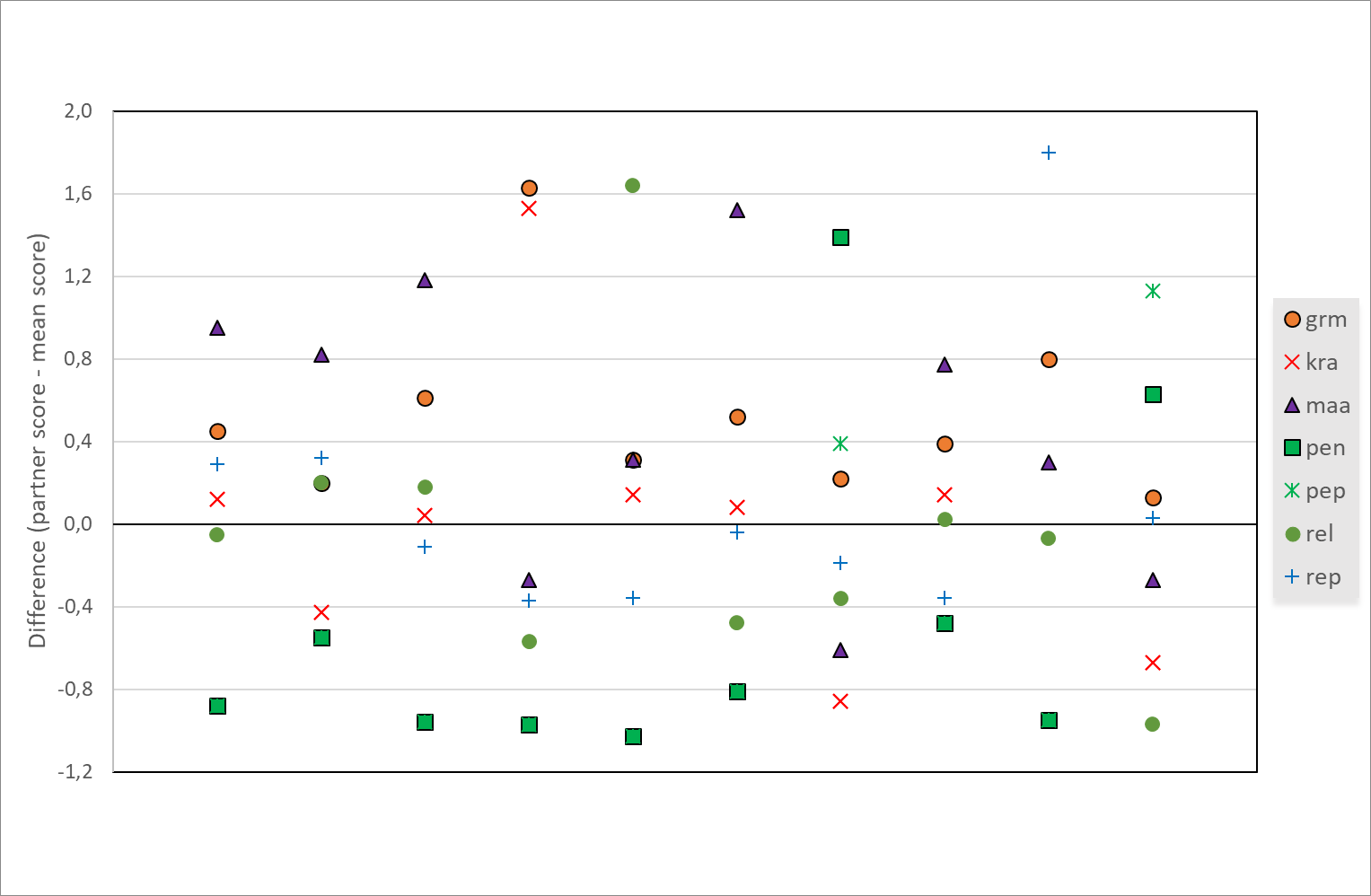

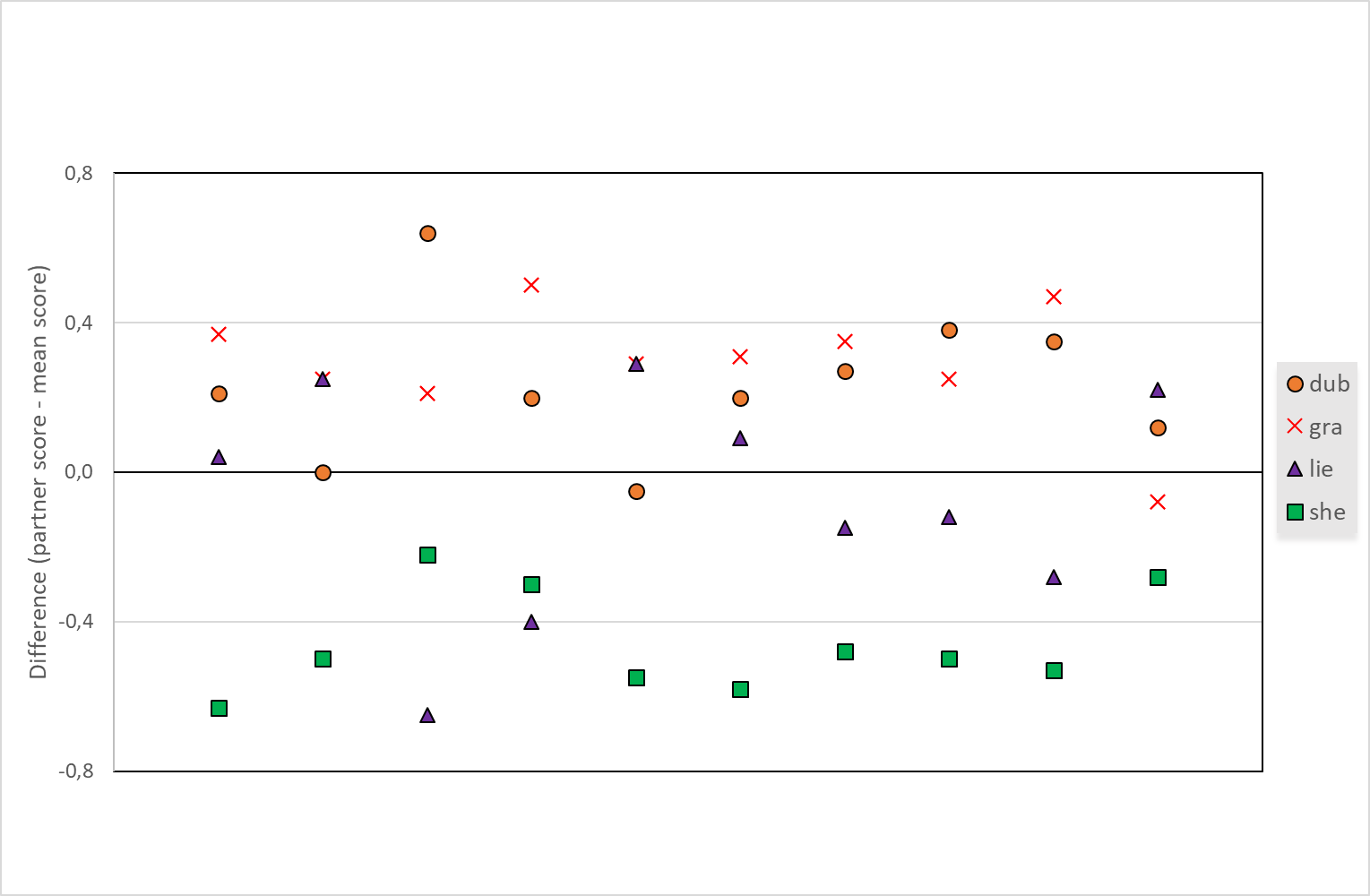


Legend : The plots show the difference of each partner answers compared with the overall mean of all partners for a given competency domain. E.g. in Year 1 and for the competency domain “collaboration and partnership” the Granada component (symbol “gra” in the graph) scored around 0.4 above the average of all 4 partners.

**Method for transforming the scores**

The table S1 below shows original (top half) and transformed (bottom half) scores for the four Year 1 partners/components.

We first averaged the original scores for each partner (column “row mean”, at the right hand side, top half of the table), as well as the overall mean original scores across partners and domains (value = 2.4). The transformed score is obtained by calculating the relative difference between the original score and the row mean and adding the result to the overall mean score. As an example the calculation for the component “Sheffield” and the first competency domain “Collaborations…” is as follows : transformed score = ((1.3 – 1.9)/1.9) + 2.4= 2.1.

The second part of the table displays the transformed scores. For each component, scores vary between competency domains in a similar way than original scores do. However, the row mean are now the same for each component.

Table S1: Original and transformed scores for Year 1 components of the Europubhealth Master

|  |  | **Competency domains** | | | | | | | | | |  |
| --- | --- | --- | --- | --- | --- | --- | --- | --- | --- | --- | --- | --- |
|  | **Partner - component** | **Collaborations and partnerships** | **Communication culture and advocacy** | **Prof. dev., ethical reflexive practice** | **Governance and ressource management** | **Law policies and ethics** | **Leadership and system thinking** | **One health and health security** | **Organisational literacy and adaptability** | **Promoting health** | **Science practice** | **row mean** |
| **Original scores** | Dublin | 2,2 | 2,6 | 3,0 | 2,2 | 2,3 | 2,7 | 2,5 | 2,9 | 2,9 | 2,9 | **2,6** |
|  | Granada | 2,3 | 2,9 | 2,6 | 2,5 | 2,7 | 2,8 | 2,6 | 2,8 | 3,0 | 2,7 | **2,7** |
|  | Liege | 2,0 | 2,9 | 1,7 | 1,6 | 2,7 | 2,6 | 2,1 | 2,4 | 2,3 | 3,0 | **2,3** |
|  | Sheffield | 1,3 | 2,1 | 2,1 | 1,7 | 1,8 | 1,9 | 1,8 | 2,0 | 2,0 | 2,5 | **1,9** |
|  | **Overall mean** |  |  |  |  |  |  |  |  |  |  | **2,4** |
|  |  |  |  |  |  |  |  |  |  |  |  |  |
| **Trans-formed scores** | Dublin | 2,2 | 2,4 | 2,5 | 2,2 | 2,3 | 2,4 | 2,3 | 2,5 | 2,5 | 2,5 | **2,4** |
|  | Granada | 2,3 | 2,5 | 2,3 | 2,3 | 2,4 | 2,4 | 2,4 | 2,4 | 2,5 | 2,4 | **2,4** |
|  | Liege | 2,3 | 2,6 | 2,1 | 2,1 | 2,5 | 2,5 | 2,3 | 2,4 | 2,4 | 2,7 | **2,4** |
|  | Sheffield | 2,1 | 2,5 | 2,5 | 2,3 | 2,3 | 2,4 | 2,3 | 2,4 | 2,4 | 2,7 | **2,4** |
|  | **Overall mean** |  |  |  |  |  |  |  |  |  |  | **2,4** |
